# Investigation of the effects of a Kiperin Bromelain & Papain complex in normal colon, mammary, and skin cells

**DOI:** 10.1038/s41598-026-52512-9

**Published:** 2026-05-21

**Authors:** Lutfiye Karcioglu Batur, Cuneyd Yavas, Nezih Hekim

**Affiliations:** 1 Department of Molecular Biology and Genetics, Faculty of Engineering and Natural Sciences, Biruni University, Merkezefendi, 75 Sk No:1-13 M. G, 34015 Zeytinburnu, Istanbul, Turkey; 2 Biruni University Research Center (B@MER), Biruni University, 34015 Istanbul, Turkey; 3 MALTA Life Science Park, COMED Therapeutics Ltd, San Gwann, Malta

**Keywords:** Bromelain, Papain, Gene expression, Wound healing, Cell proliferation, Biotechnology, Cell biology, Diseases, Molecular biology

## Abstract

Bromelain is a proteolytic enzyme complex naturally found in the stem, fruit, and juice of pineapple, while papain is a proteolytic enzyme derived from the latex of the papaya plant. These bioactive components have attracted increasing scientific interest due to their reported roles in cell proliferation and wound healing, suppression of oxidative stress through free radical scavenging, and apoptosis-related pathways. Although bromelain and papain have been associated with various health-related effects, their effects on healthy cells remain largely unexplored. In this study, different concentrations of Kiperin Bromelain & Papain (KBP) (1.0–3.5 µg/mL) were tested in healthy cell lines. Based on the concentration showing the highest cell viability, 2.5 µg/mL was selected for further analyses, and CCD-18Co (colon fibroblasts), HME-1 (mammary epithelial cells), and HaCaT (keratinocytes) were treated accordingly. Cell viability was assessed by cell counting, and wound healing was evaluated. Gene expression levels of *MMP9*, *IVL*, *FLG*, *IL-6*, *IL-10*, *NFKβ*, and *TNF-α* were measured by real-time PCR. KBP exposure increased cell viability in all cell lines, with the most pronounced effect observed at 2.5 µg/mL. Wound-healing assays indicated changes in wound closure following KBP exposure. In addition, KBP treatment was associated with decreased expression of inflammation-related genes (*IL-6*, *IL-10*, *NFKβ*, and *TNF-α*) and increased expression of genes related to skin barrier/keratinization (*IVL* and *FLG*). The magnitude of these effects differed across cell types, indicating cell-specific responses. Further research is warranted to validate these findings in in vivo models and to clarify the potential applications of KBP in dermatological contexts.

## Introduction

Research on the biological activities of bromelain and papain, and their capacity to regulate cellular processes and gene expression in vitro, has gained increasing attention due to their broad range of potential applications. Bromelain is a complex mixture of proteolytic enzymes derived from pineapple, whereas papain is a cysteine protease obtained from papaya. Both enzymes have been investigated for their roles in modulating inflammation and supporting tissue repair–related processes, including wound-healing responses, alongside other reported bioactivities^1–5^.

Over the past decades, research has progressed from descriptive studies of enzymatic activity to more detailed investigations into molecular mechanisms across different cell models^6–8^. In this context, bromelain and papain have been associated with regulatory effects on cellular pathways linked to inflammation, apoptosis, and gene expression modulation^9–11^. However, the extent to which these enzymes influence specific gene expression programs and related cellular responses remains incompletely characterized, particularly across different cell types^6,7,12^. In addition, the comparative effects of bromelain and papain, as well as their potential combined activity, remain subjects of ongoing investigation^6,13–16^. Clarifying these mechanisms is important for improving biological understanding and informing the design of future applications.

Conceptually, prior studies have linked the biochemical properties of bromelain and papain as proteases with regulatory roles in cellular processes such as inflammation and apoptosis, as well as gene expression modulation^4,17,18^. These enzymes have been discussed in relation to key signaling pathways that govern cellular survival, proliferation-related responses, and immune regulation, including *NFKβ*, MAPK, and AMPK^12,19^. A clearer understanding of these relationships is needed to interpret how bromelain and papain containing formulations may shape cellular behavior in different in vitro settings.

Although many studies have focused on cancer or disease-related models, direct data regarding the effects of bromelain and papain on the expression of genes associated with inflammation-related responses and skin barrier–related markers in healthy human cell lines remain limited. Therefore, this study aimed to investigate the in vitro responses of healthy human cell lines following treatment with Kiperin Bromelain & Papain (KBP). Specifically, we evaluated KBP-associated changes in cell viability, wound closure in a wound-healing assay, and the expression of selected genes related to inflammation and skin barrier/keratinization (*MMP9*, *IVL*, *FLG*, *IL-6*, *IL-10*, *NFKβ*, and *TNF-α*). By focusing on non-cancerous, physiologically normal human cells, this work seeks to contribute to a more balanced and context appropriate understanding of bromelain and papain related cellular effects in vitro.

## Materials and methods

The commercial product KBP, used as the test compound, which was supplied by Kiperin Pharmaceutical Food Industry and Trade Limited Company (Istanbul, Turkiye). KBP consists of Bromelain (2500 GDU) and Papain extract. The bromelain and papain used in this study were supplied as a nutraceutical-grade, plantderived enzyme complex (Kiperin Bromelain & Papain, Kiperin Pharmaceutical, Turkiye). The bromelain component is naturally extracted from pineapple stem and fruit, and the papain component is extracted from papaya latex. Kiperin Bromelain & Papain was selected for this study because it is a widely used nutraceutical-grade enzyme complex that is commercially accessible and frequently consumed by the general population without medical supervision.

The trials were performed in a randomized and blinded fashion, with three independent repetitions. Among the doses tested, concentrations of 1 µg/mL, 1.5 µg/mL, 2 µg/mL, 2.5 µg/mL and 3 µg/mL and 3.5 µg/mL were determined. This concentration range was determined by considering the studies in the literature and further studies were performed with the concentration that caused the best cell increase^13,19,20^. These doses were determined by three independent cell counts using the Cell Viability Detection Kit-8 (CVDK-8; Eco Tech Biotechnology, Turkiye) and Thoma counting chamber. Selected doses were normalised to in vitro cell counts (6 × 10^4^ cells/well) to approximate physiologically achievable levels while minimising off-target cytotoxic responses. The common concentration most effective at proliferation in healthy human cells was used. To evaluate the cytotoxic response and validate the sensitivity of the assay system, hydrogen peroxide (H₂O₂) was used as a positive control. H₂O₂ is a well-established oxidative agent that induces reactive oxygen species (ROS) generation, leading to cellular stress, membrane damage, and apoptosis. For this reason, it is widely used as a positive control in cell viability and proliferation assays to confirm the effectiveness of the experimental setup. In this study, a concentration of 1.6 mM, which is a high lethal dose for 293T cancer cells reported in the literature, was used^21^.

An initial dose-range screening was performed using KBP at 1.0–3.5 µg/mL in CCD-18Co, HME-1, and HaCaT cells. The working concentration for downstream experiments was selected a priori based on the dose that produced the highest viability/metabolic activity readout while remaining within the tested non-cytotoxic range. Across the screened doses, 2.5 µg/mL yielded the highest viability signal in all three cell lines. Therefore, this concentration was used for subsequent wound-healing and gene expression analyses. The purpose of this selection was to evaluate KBP-associated responses in healthy cells under conditions compatible with maximal viability.

Downstream analyses were performed at a single time point (24 h), selected based on the wound-healing assay, where closure was near-complete by 24 h. Accordingly, time-dependent effects beyond this interval were not assessed.

The findings of this study are limited to mRNA-level gene expression changes. Since protein-level validation and functional assays were not performed, the results should be interpreted cautiously and should not be considered direct evidence of protein-level or functional effects.

### Cell culture

The present study was approved by the Scientific Research Ethics Committee of Biruni University on July 28, 2025, under decree number 2024-BIAEK/12-23. CCD-18Co human colon fibroblast cells from ATCC (CRL-1459), HME-1 human breast epithelium form ATCC (CRL-4010) and HaCaT human skin cells from Cytion (Product number: 300493) were used in this study. Cells were cultured in Dulbecco’s Modified Eagle Medium (DMEM, ThermoFisher Scientific) supplemented with 10% fetal bovine serum (FBS, Thermo Fisher Scientific) and 1% penicillin–streptomycin (Thermo Fisher Scientific) and maintained at 37 °C in a humidified incubator with 5% CO₂ (WCI-180, Wiggens,

Germany) throughout the experiments. Wound healing and viability-proliferation were performed as two separate experimental studies conducted at different time points. Each experiment was repeated three times independently to ensure reproducibility and to evaluate each parameter individually. All experiments were conducted in three independent biological replicates with three technical replicates per sample. Cell viability-proliferation assay and qRT-PCR for expression levels of inflammation-oedematous markers results were obtained after 24 h of KBP exposure. In the wound healing study, cells were monitored at 4-h intervals and the experiment was terminated when complete closure was observed.

### Cell viability and proliferation assay

Cell viability following KBP treatment was evaluated using the WST-8 colorimetric method with the Cell Viability Detection Kit-8 (CVDK-8; EcoTech Biotechnology, Turkiye). A total of 3 × 10^3^ cells were seeded into each well of 96-well plates in triplicate for all groups. To ensure that an equal number of cells were seeded into each well, cells were counted using a Thoma counting chamber prior to plating. After incubation for 24 h under standard culture conditions, the control and treatment groups were prepared by administering KBP to the designated wells. Cell counting and viability analysis were performed using a Thoma counting chamber. Cells from each experimental group were counted separately, and the mean cell number and viability percentage were calculated. The data were statistically evaluated based on replicate measurements (One-way ANOVA).

Each cell line was assigned into two groups: untreated control and KBP-treated groups receiving increasing concentrations of the compound (1–3.5 µg/mL). Following 24 h of exposure, 10 μL of WST-8 reagent was added to each well. After an additional 3 h incubation at 37 °C, absorbance was measured at 450 nm using a BioTek Epoch Microplate Spectrophotometer (Epoch, USA).

### Wound healing assay

To examine the effects of KBP supplement treatment on wound healing, cells were seeded in a sufficient number of wells at 4 × 10^5^ per well in 6-well plates. A wound healing (scratch) assay was performed using KBP at a concentration of 2.5 µg/mL. For each cell line, cells were divided into two experimental groups: a negative control group (untreated) and a KBP-treated group (2.5 µg/mL). Confluent monolayers (≥ 80%) of CCD-18Co, HME-1 and HaCaT cells were scratched using a sterile 100 µL pipette tip to create a uniform wound. Images of the wound area were captured at 0, 12 and 16 h (based on cell line) post-scratch using the ZOE Fluorescent Cell Imager, con sistently positioned at the same marked field of view for each time point. The amount of closure in the wound area and the density of cells migrating to the wound area were evaluated according to the study that was conducted by Abuaisha et al. and Jonkman et al.^22,23^. Wound closure area was measured using Image J (1.54 g NIH, USA) programme. We analysed the selected areas of interest using photographs taken with the cell imager (ZOE) at 0, 12 and 16 h. With the help of our addon, we determined the scratch area, the wound coverage ratio of the total area, and the mean and standard deviation of the scratch width. We calculated the wound closure percentage based on the 0th hour using the “wound healing area (%) = [(M0 – Mh) / M0] × 100” equation (M0 is the wound area at 0th, and Mh the wound area at the relevant hour). For each cell line, cells were divided into two experimental groups as negative control (untreated) and treated with 2.5 µg/mL concentration of KBP.

### qRT-PCR for expression levels of inflammation and edematous markers

In 6-well plates, 4 × 10^5^ cells of each cell line were seeded. After 24 h cells were treated as designed. 24 h later cells were collected for RNA isolation. Total RNA was extracted from cells using the EZ-10 Total RNA Miniprep Kit (Biobasic, Canada) according to the instructions provided by the manufacturer. The concentration and purity of the isolated RNA were evaluated using a NanoDrop 2000 microvolume spectrophotometer (Thermo Fisher Scientific, USA). A total of 300 ng of RNA was converted into complementary DNA (cDNA) using the OneScript Plus cDNA Synthesis Kit (Applied Biological Materials Inc., Richmond, Canada), following the manufacturer’s instructions. The reverse transcription reaction was carried out in a T100 Thermal Cycler (BioRad, Singapore) at 52 °C for 15 min.

Quantitative real-time PCR (qPCR) analysis was conducted using the BlasTaq 2X qPCR MasterMix (Applied Biological Materials Inc.), following the guidelines provided by the manufacturer. Each reaction contained 2 µL of cDNA template, 0.5 µL of each primer (10 µM), and 10 µL of Master Mix, with a final volume adjusted to 20 µL using nuclease-free water. The forward and reverse primer sequences used in the study are listed in Table 1. The primers were designed using the NCBI Primer-BLAST program. Gene-specific sequences were used as input, and primers were selected based on optimal GC content, melting temperature, and amplicon size. Primer specificity was verified against the human genome database to avoid off-target amplification. All primers were tested at a range of annealing temperatures, and the reactions were performed using the empirically determined optimal annealing temperature for each primer pair. PCR amplification and fluorescence detection were carried out using the Bio-Rad CFX96 Real-Time PCR System. The thermal cycling program consisted of an initial denaturation at 95 °C for 3 min, followed by 45 cycles of denaturation at 95 °C for 15 s, annealing at 60 °C for 15 s, and extension at 72 °C for 15 s. Fluorescence signals were measured at the completion of each extension phase. Gene expression levels were calculated using the 2^−ΔΔCq^ method, with *GAPDH* used as the reference housekeeping gene^24^

**Table 1:** Forward and reverse primer sequences of the studied genes.

Gene	Forward primer	Reverse primer
*MMP9*	5′-CCCTGGAGACCTGAGAACCA-3′	5′-CCCGAGTGTAACCATAGCGG-3′
*IVL*	5′-GGTCCAAGACATTCAACCAGCC-3′	5′-TCTGGACACTGCGGGTGGTTAT-3′
*FLG*	5′-GCTGAAGGAACTTCTGGAAAAGG-3′	5′-GTTGTGGTCTATATCCAAGTGATC-3′
*IL-6*	5′-GGCACTGGCAGAAAACAACC-3′	5′-CCAGGCAAGTCTCCTCATTG-3′
*IL-10*	5′-CCAGGGCACCCAGTCTGAGA-3′	5′-AGCTTGGGGCATCACCTCCT-3′
*NFKβ*	5′-TACGATGGAACCACACCCCTG-3′	5′-TCTGCTCCTGCTGCTTTGAGA-3′
*TNF-α*	5′-CAGCCTCTTCTCCTTCCTGAT-3′	5′-GCCAGAGGGCTGATTAGAGA-3′
*GAPDH*	5′-CCACCCATGGCAAATTCC-3′	5′-GGGATTTCCATTGATGACAAG-3′

### Statistical analysis

All statistical analyses were performed using GraphPad Prism software (version 10.4.1). Data are presented as mean ± standard deviation (SD). Since all experiments were conducted at a single fixed timepoint, no time-dependent analyses were performed. For comparisons involving more than two groups, a one-way ANOVA was applied to evaluate differences among the experimental conditions, followed by Tukey’s post hoc test when appropriate. For comparisons between two independent groups (control and KBP-treated), an unpaired t-test was used. Each figure legend has been updated to specify the exact statistical test applied to the corresponding dataset. A *p*-value of < 0.05 was considered statistically significant (*p < 0.05, **p < 0.01, *p < 0.001, ns: not significant).

## Results

### Cell viability and proliferation

**Figure 1:**
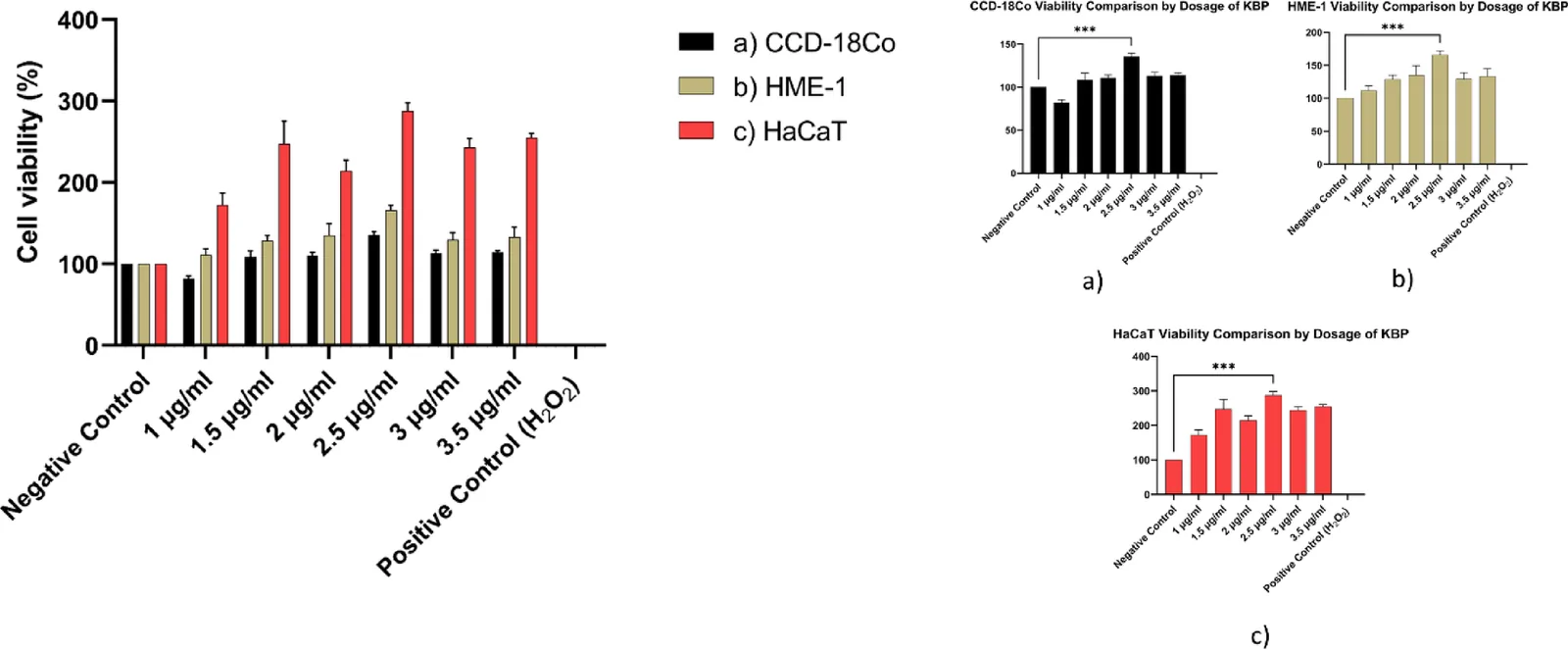
Treatment led to an increase in the number of CCD-18Co, HME-1 and HaCaT cells treated with KBP (1–3.5 µg/mL). The best concentration in 3 cell lines [(**a**) CCD-18Co, (**b**) HME-1, and (**c**) HaCaT cells were treated with KBP at the indicated concentrations] was 2.5 µg/mL (***p < 0.001).

**Figure 2:**
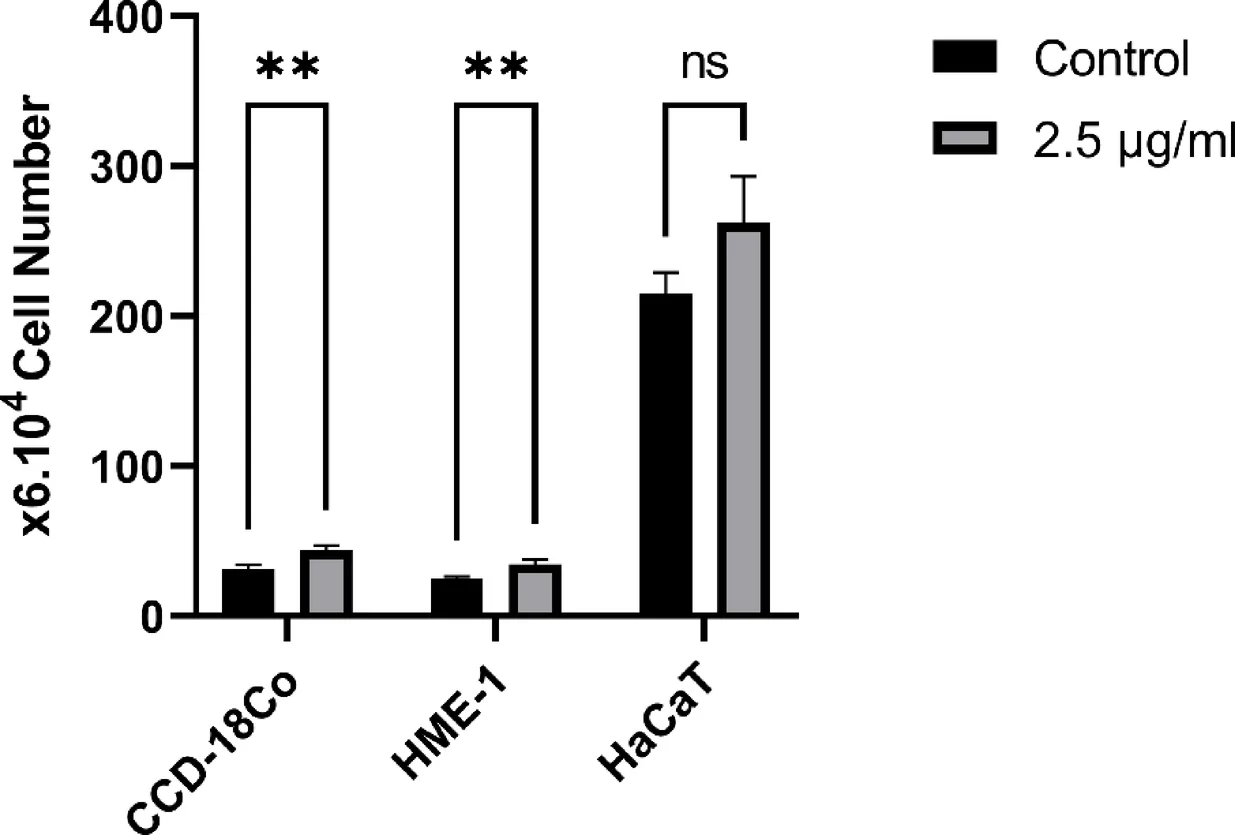
Effect of 2.5 µg/mL KBP treatment on cell proliferation in CCD-18Co, HME-1 and HaCaT cell lines. Bar graphs represent mean ± standard deviation (SD) of three independent experiments. (**p < 0.01 vs. control group, ns = no significant).

ELISA absorbance measurements following the CVDK-8 assay revealed that untreated negative control groups showed similar results to KBP-treated cells (absorbance was measured at 450 nm using a BioTek Epoch Microplate Spectrophotometer (Epoch, USA)). Based on the dose–response analysis presented in Fig. 1, KBP treatment increased cell viability in all three normal human cell lines, with the response reaching an upper level at 2.5 µg/mL. Therefore, 2.5 µg/mL was selected as the lowest effective dose providing the observed upper-level response across all tested normal cell lines and was used in subsequent experiments (viability rates of 152.7% in HME-1, 122.2% in CCD18-Co, and 274.1% in HaCaT cells). For further investigations, all three healthy human cell lines were evaluated under control and KBP-treated conditions at 2.5 µg/mL (Fig. 2). A significant elevation in cell numbers was observed in CCD-18Co and HME-1 cells following KBP treatment compared to the control group (**p < 0.01). Although an increase was observed in the HaCaT cell line, no statistical significance was detected. These results indicate that KBP exerts a stimulatory effect on the proliferation of normal human cells.

### Assessment of wound healing

**Figure 3:**
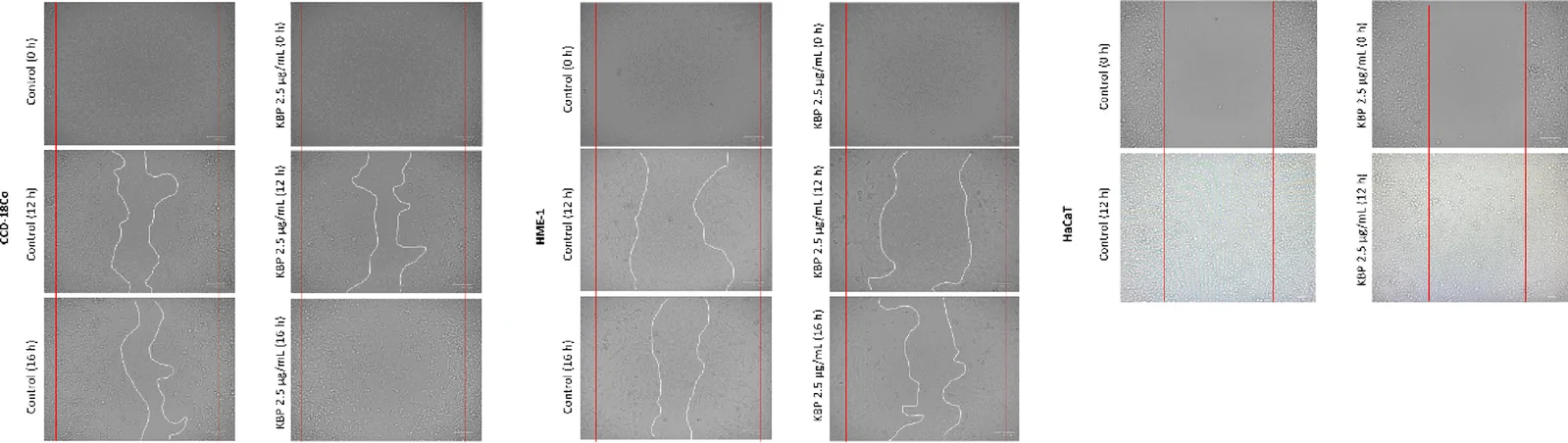
Scratch wound assay for evaluating the wound healing of CCD-18Co, HME-1 and HaCaT cell lines 0, 12 and 16 h after 2.5 μg/mL of KBP treatment.

According to the wound healing assay results, treatment with 2.5 μg/mL of KBP significantly enhanced the wound healing of CCD-18Co cell compared to both the non-treated control group (Fig. 3). When CCD-18Co human colon fibroblast cells were examined for 16 h, wound healing was faster in cells treated with KBP compared to the control group. HME-1 human breast epithelium cells showed partially better wound closure compared to control (Table 2). Since HaCaT human skin cells healed faster than the other two cell lines, a complete comparison could not be performed.

**Table 2:** The wound healing (%) of the cell scratch assay.

Groups	Incubation Hours	Wound Healing Rate of CCD-Co18 Cells	Wound Healing Rate of HME-1 Cells	Wound Healing of HaCaT Cells
Control	12th	79.74%	55.78%	100%
2.5 µg/ml KBP	12th	79.48%	52.74%	100%
*p*-value		0.7746	0.8759	–
Control	16th	84.76%	73.28%	–
2.5 µg/ml KBP	16th	93.56%	76.73%	–
*p*-value		< 0.001	0.3325	–

*p*-values indicate the statistical significance of differences in the percentage of wound healing between control and KBP-treated groups. *p* value < 0.05 was considered statistically significant. Hydrogen peroxide (H₂O₂) treatment resulted in complete loss of cell viability. Therefore, quantitative values were not included in the table. In addition, because no proliferation-blocking agent was used in the wound-healing assay, the observed wound closure was interpreted relative to the negative control under identical experimental conditions.

### Expression levels of inflammation and edematous markers

**Figure 4:**
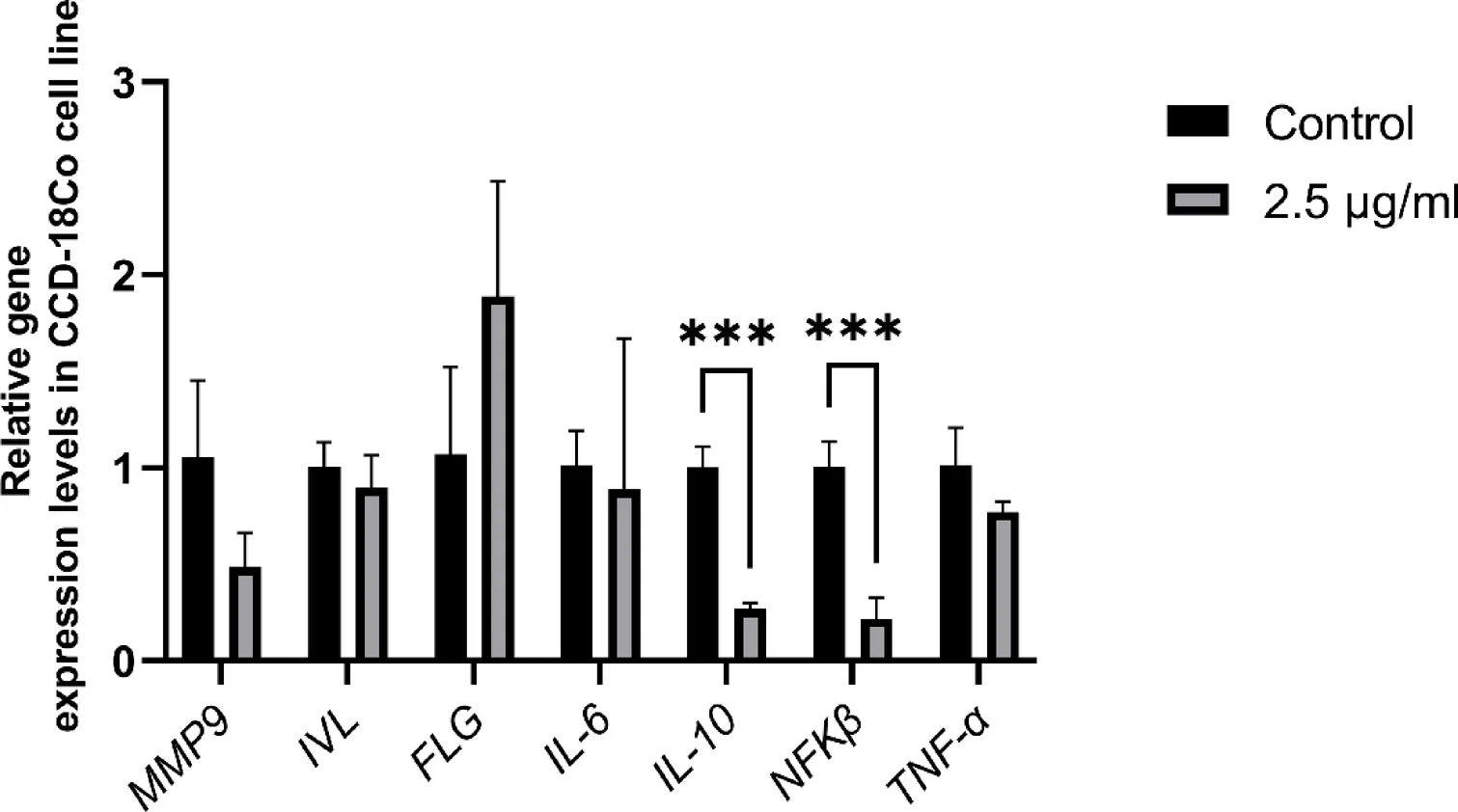
The mRNA expression levels of *MMP9*, *IVL*, *FLG*, *IL-6*, *IL-10*, *NFKβ*, and *TNF-α* were evaluated in CCD-18Co cells following KBP treatment. The data, expressed as mean ± standard deviation (SD), were obtained from three independent experiments. While no statistically significant differences were detected for *MMP9*, *IVL*, *FLG*, *IL-6*, and *TNF-α* between the control and KBP-treated groups, a marked reduction in *IL-10* and *NFKβ* expression was observed (***p < 0.001, appendix was added only to those that were significant).

**Figure 5:**
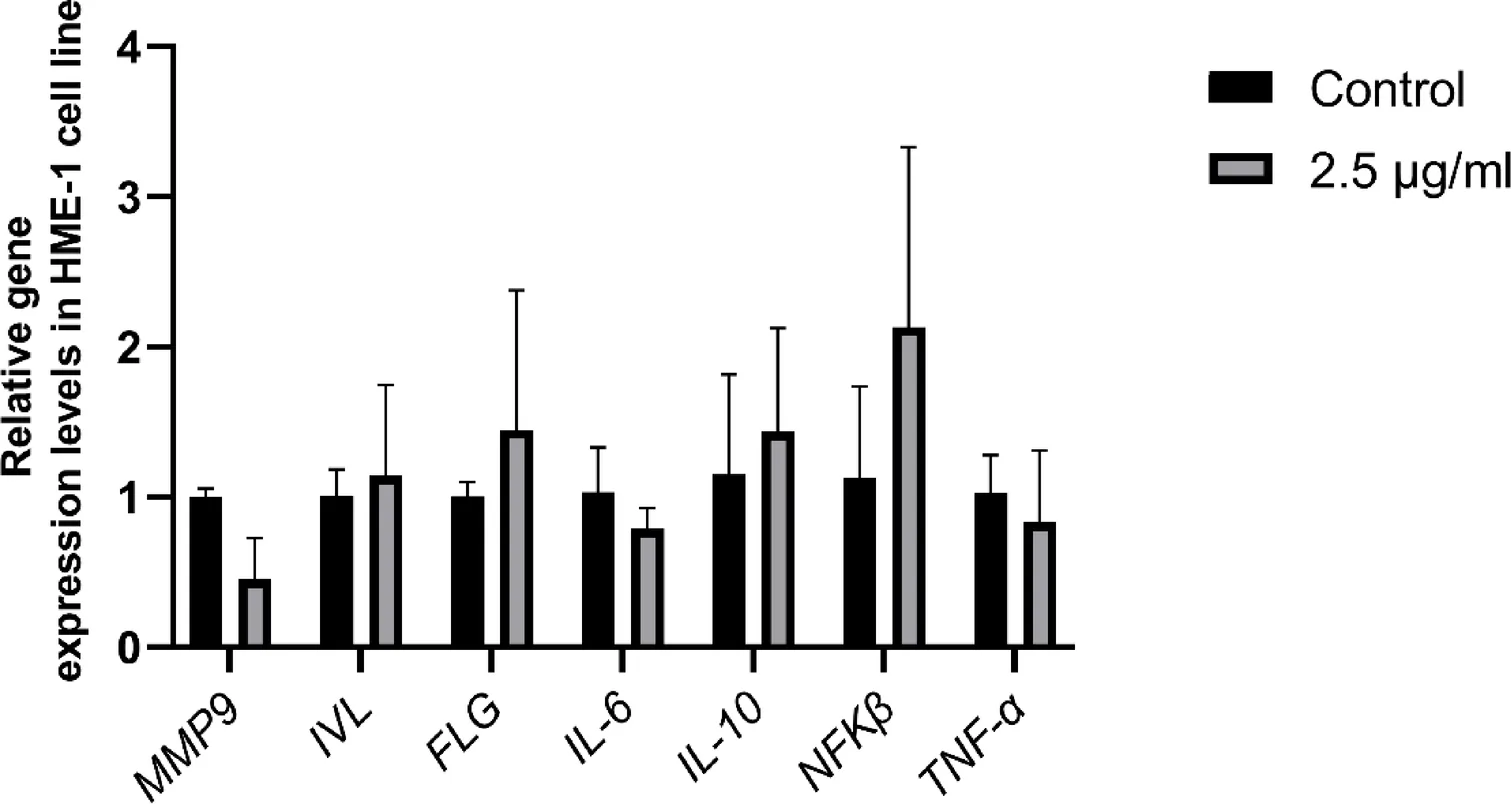
*MMP9*, *IVL*, *FLG*, *IL-6*, *IL-10*, *NFKβ* and *TNF-α* expression levels in HME-1 cells follow-ing KBP treatment. Data are presented as mean ± standard deviation (SD) from three independent experiments. Statistical analysis showed no significant difference between treated and control groups in both cell lines (Appendix was added only to those that were significant).

The expression levels of *MMP9* (matrix metalloproteinase-9, is extracellular matrix–degrading enzyme involved in tissue remodeling and inflammation), *IVL* (Involucrin is differentiation marker involved in epidermal barrier formation), *FLG* (Filaggrin is key structural protein essential for epidermal barrier integrity.), *IL-6* (Interleukin-6 is pro-inflammatory cytokine involved in immune and inflammatory signaling), *IL-10* (Interleukin-10 is anti-inflammatory cytokine that regulates immune responses), *NFKβ* (Nuclear factor kappa light chain enhancer of activated B cells is transcription factor that controls the expression of inflammation-related genes) and *TNF-α* (Tumor necrosis factor alpha is major pro-inflammatory cytokine involved in immune regulation and cell signaling) in CCD-18Co, HME-1 and HaCaT cells under control conditions and after treatment with 2.5 μg/mL KBP are shown. In HME-1 cells, KBP treatment was associated with a slight increase in *IVL* and *FLG* mRNA expression; however, these differences were not statistically significant (p > 0.05). The observed trends reflect biological modulation rather than a definitive regulatory effect at the tested concentration. For CCD-18Co cells, *MMP9*, *IL-6*, *IL-10*, *NFKβ* and *TNF-α* expression decreased with KBP complex treatment. Conversely *FLG* gene expression was increased. For HME- 1 cells, *MMP9*, *IL-6* and *TNF-α* expression decreased following KBP treatment, while *IVL*, *FLG*, *IL-10* and *NFKβ* expression increased (Figs. 4, 5).

**Figure 6:**
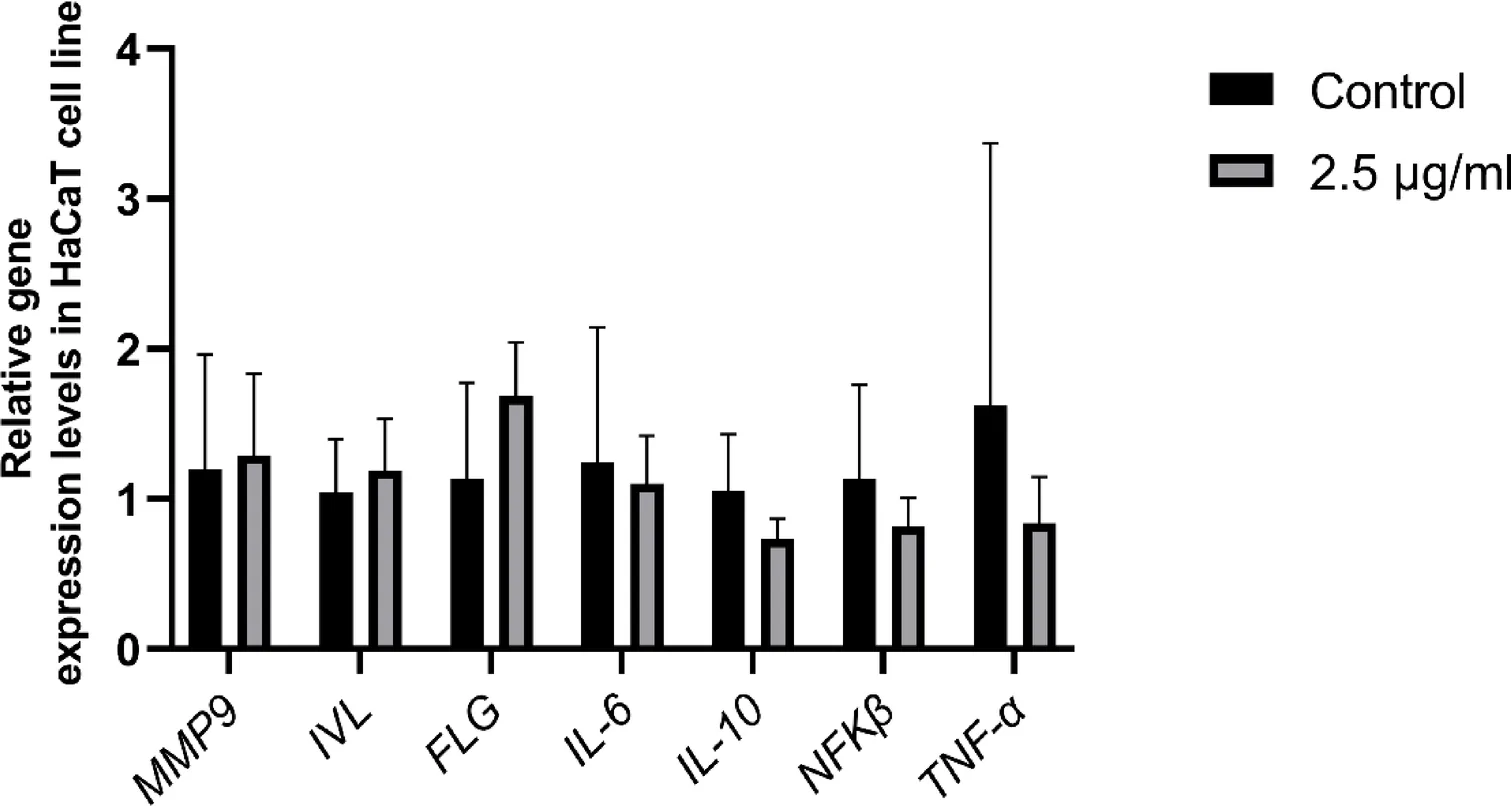
*MMP9*, *IVL*, *FLG*, *IL-6*, *IL-10*, *NFKβ* and *TNF-α* expression levels in HaCaT cells follow-ing KBP treatment. The results, expressed as mean ± standard deviation (SD) from three inde-pendent experiments, indicated that there was no statistically significant difference between the KBP-treated groups and the untreated controls in either of the cell lines (Appendix was added only to those that were significant).

For HaCaT cells, the expression levels of *IL-6*, *IL-10*, *NFKB*, *TNF-α*, *FLG*, *MMP9*, and *IVL* remained largely unchanged following KBP treatment, with no statistically significant differences observed between control and treated groups (Fig. 6). These results show that 2.5 μg/mL KBP causes changes in the levels of genes such as *MMP9*, *IVL*, *FLG*, *IL-6*, *IL-10*, *NFKβ* and *TNF-α* in the regulation of inflammation and edema in different cell lines. In CCD-18Co cell line, HME-1 cell line and HaCaT cell lines, inflammation pathways were decreases and changes in functional genes in skin barriers were observed. This highlights the potential role of KBP in modulating the regulation of inflammation and edema.

## Discussion

Importantly, the selected concentration did not induce cytotoxicity in any of the tested cell lines, suggesting that the observed gene expression changes reflect a physiological rather than a stress-induced transcriptional response^25^. Our results show that the KBP complex at a dose of 2.5 µg/mL caused a statistically significant increase (**p < 0.01) in cell numbers in CCD-18Co and HME-1 cell lines compared to the control group, whereas no statistically significant change was observed in HaCaT cells. This finding suggests that KBP suggests activating effects on the growth of healthy human cells. In the literature, it is widely reported that these enzymes inhibit the growth of cancer cells and even lead to apoptosis^15,20,21^. The inclusion of multiple non-malignant cell lines was intentionally designed to evaluate the consistency and variability of KBP effects across distinct cellular contexts, rather than to address a single tissue-specific hypothesis. Therefore, the findings should be interpreted individually within the specific biological context of each cell type.

In our study, KBP did not induce antiproliferative effects in healthy cell lines, contrary to what has been reported in various cancer models. Studies in the literature have reported a cancer-specific cytotoxic mechanism^6–9^, and due to the limited number of studies in the literature on healthy cellular systems, the importance of evaluating bromelain and papain in non-malignant cells is emphasised. Therefore, our findings provide preliminary data on the cellular responses and safety profile of KBP in normal human cell lines, which is an important step to take before evaluating potential therapeutic applications. However, a proliferation-promoting effect in healthy cells may suggest a potential role of KBP in processes such as tissue repair or regeneration, an area rarely emphasised in the literature. These results suggest that the inflammation-modulating activity observed in vitro may vary depending on the cellular context and physiological state. In previous studies examining nutrient and supplement-based formulations in different cellular systems, the reported biological responses also varied depending on cell type and physiological state^26–28^. The selection of 2.5 µg/mL as the working concentration was based on dose–response analyses suggesting the highest level of cell viability across all tested healthy cell lines. This approach was intentionally adopted to evaluate the cellular and molecular responses under conditions of optimal cellular activity and viability. In the context of non-diseased cell models, selecting a concentration that supports maximal viability represents a biologically relevant condition rather than a confounding factor, as it allows the assessment of responses in a physiologically favorable environment^29^ Therefore, the observed gene expression and wound healing changes are interpreted within this context. Also the use of multiple healthy human cell lines representing different tissue types (colon fibroblasts, mammary epithelial cells, and keratinocytes) was intentionally designed to evaluate the consistency of the observed molecular and functional responses across diverse cellular environments. Rather than focusing on a tissue-specific effect, this approach allows the identification of common response patterns and provides insight into the general biological activity of the compound under physiologically relevant conditions.

In CCD-18Co cells, KBP significantly reduced *IL-10* and *NFKβ* mRNA levels (*p* < 0.05). For *MMP9, IL-6*, *FLG*, and *TNF-α*, mean expression values were lower in the KBP group; however, the differences were not statistically significant (*p* > 0.05). This finding is consistent with previous literature reporting that bromelain inhibits *NFKβ* activity and has been associated with reduced expression of pro-inflammatory cytokines such as *IL-6* and *TNF-α*^30–32^. The observed downregulation of these inflammatory markers in healthy colon fibroblasts suggests that KBP exposure may be associated with modulation of inflammation-related markers without affecting cell viability. Furthermore, the increase in *FLG* expression may be associated with barrier or structure related cellular responses, further suggesting a potential association with cellular processes related to tissue homeostasis. The decrease in *MMP9* also indicates that inflammation is suppressed, because *MMP9* plays a key role in inflammatory processes by degrading the extracellular extra cellular matrix^22,23^. These results suggest that KBP exposure is associated with transcriptional changes in inflammatory gene expression in colonic fibroblasts, which are consistent with patterns reported in the literature. Although the observed downregulation *of IL-10* may appear unexpected given its well-known anti-inflammatory role, this finding may reflect alterations in the broader inflammatory gene expression profile following KBP exposure in non-diseased colon cells. In the absence of strong pro-inflammatory stimuli, *IL-10* expression might be reduced as part of a balanced immune response, potentially reflecting a balanced cellular response under the tested conditions^31,32^.

Also *FLG* gene and their proteolysed amino acids are multifunctional protein components that contribute to the formation of an intact stratum corneum barrier^33^. The increase in *FLG* gene expression may be associated with barrier-related cellular responses. These findings suggest that KBP exposure may be associated with changes in markers related to tissue integrity and inflammation-associated responses. In addition, wound healing methods are another method that shows the effects of the applied substances on cell lines^1,34,35^. KBP treatment was associated with increased wound closure in CCD-18Co cells after 16 h of incubation compared to the control group. This finding suggests that KBP may be effective in fibroblast-based wound healing processes possibly associated with altered wound healing behaviour of connective tissue cells.

In studies on skin health, it has been reported that mutations in the *FLG* gene of patients with atopic dermatitis significantly reduce the expression of this gene^36,37^. In another study, it was shown that some plant extracts significantly increased *FLG* and *IVL* gene expressions^38^. Previous studies have shown that certain plant extracts can upregulate *FLG* and *IVL* expression, contributing to improved skin barrier function. In line with these findings, KBP treatment in our study also increased FLG and IVL expression, suggesting a potential supportive role of KBP in skin barrier-related cellular functions. Consistent with our findings, several studies have reported that natural proteolytic and plant-derived extracts exert anti-inflammatory and antioxidant effects in skin cells, supporting their role as promising protective effect candidates^39–41^. According to our findings, KBP increased *FLG* and *IVL* expression levels in cell lines, suggesting a potential role in supporting barrier-related cellular functions and modulating inflammation-associated responses in vitro. Although *IVL* and *FLG* expression showed a modest upward tendency in HME-1 cells, the changes did not reach statistical significance; therefore, no conclusion regarding skin barrier protein synthesis can be drawn based on the current data. In the literature, it has been reported that proteases such as bromelain and papain have anti-inflammatory effects and can increase *IL-10* anti-inflammatory cytokines^30,34,42^. The slight increase in *IL-10* expression observed in our study suggests that KBP may suggest inflammation-modulating activity^43,44^. In parallel with these reports, KBP exposure in HME-1 cells was associated with reduced expression of inflammation-related markers and increased expression of barrier-associated genes (*IVL*, *FLG*). Moreover, cell promote cell proliferation status was also observed in both cell viability, wound healing and live cell counts (Figs. 1, 2, 3). Treatment of HME-1 cells with KBP promoted partial wound closure compared to the control group. These findings suggest a limited cellular response associated with KBP exposure in epithelial cells.

As reported in the literature, previous studies have shown that different cell lines suggest distinct gene expression responses to the same stimulus. These differences are thought to arise from variations in their tissue of origin, genetic and epigenetic backgrounds, baseline signaling pathway activities, and cell type–specific sensitivities to chemical or radiation exposure^45–48^. The absence of statistically significant gene modulation in HME-1 and HaCaT cells may be attributed to cell line-specific responsiveness and baseline expression levels. Although the expression changes did not reach statistical significance, the directional trends observed suggest a potential modulatory effect of KBP that may become more evident at different concentrations or exposure durations. Importantly, the most significant change was detected in the CCD18-Co cell line, suggesting that the compound supports tissue-specific activity. In HaCaT cells, *IL-6*, *IL-10*, *NFKβ* and TNF-α expression decreased, *FLG* gene expression increased and *MMP9* and *IVL* expressions slightly increased with KBP treatment. The observed decrease in *IL-6* and *TNF-α* levels suggests a potential role of KBP in the modulation of inflammation-associated pathways in keratinocytes. The increase in *FLG* expression and the slight increase in *IVL* suggest a potential association with barrier-related cellular processes. Although the change was not statistically significant, the observed slight trend in *MMP9* expression may partially differ from the effects of bromelain reported in the literature, where bromelain is generally associated with reduced *MMP9* expression under inflammatory conditions^6^. These observations suggest that *MMP9* expression in healthy keratinocytes may relate to physiological processes such as wound healing or tissue remodelling, although the contribution of KBP requires further investigation. The decrease of *NFKβ*, in contrast to the increase in HaCaT cells, is more consistent with the findings of *NFKβ* inhibition in the literature. Since wound healing in HaCaT cells occurs quite rapidly, it was difficult to compare the effect of KBP with the control. However, this potential for rapid proliferation and wound healing suggests the sensitivity and suitability of keratinocytes in wound healing models. For this, observation at more frequent time intervals will provide higher resolution results.

Our results provide initial gene-level signals suggesting potential effects of KBP on inflammation and barrier-related pathways; however, protein-level analyses and functional assays will be necessary to draw definitive conclusions about inflamation and skin barrier function. However, certain limitations should be acknowledged to guide future research. As with all in vitro studies, the complexity of whole organism responses, including immune and vascular interactions, could not be fully captured. Additionally, incorporating more frequent observation intervals and additional cell lines in future studies may enhance the resolution and generalizability of the results. Despite these considerations, our findings lay a strong foundation for further exploration of KBP’s homeostasis-supporting potential effect.

## Conclusions

This study suggests that KBP is associated with measurable but cell type–dependent changes in viability, wound closure, and gene expression in three non-diseased human cell lines (CCD-18Co, HME-1, and HaCaT). KBP treatment did not show cytotoxicity at 2.5 μg/mL, which may support its tolerability in non-malignant cells under the tested in vitro conditions. Observed changes in inflammation-related genes (*IL-6*, *IL-10*, *TNF-α*, *NFKβ*) and skin barrier–associated markers (*FLG*, *IVL*, *MMP9*) indicate transcriptional modulation following KBP treatment. These findings are based on gene expression data, and the evaluation of downstream biological and functional effects will be addressed in future studies. These results provide preliminary insights into the transcriptional response of healthy cells to KBP, an area that remains underexplored in the literature. Further studies incorporating protein-level validation and functional assays are required to clarify the biological significance of these observations.

## Data Availability

All data generated or analyzed during this study are available from the corresponding author upon reasonable request.
